# Frequency and risk factors of malabsorption in patients presenting at tertiary care hospital, Karachi

**DOI:** 10.12669/pjms.40.3.7957

**Published:** 2024

**Authors:** Mohammad Masood, Riaz Hussain Channa, Nazish Butt

**Affiliations:** 1 Dr. Mohammad Masood, MBBS, FCPS (Gastroenterology & Hepatology). Assistant Professor, Department of Gastroenterology & Hepatology Jinnah Postgraduate Medical Center, Karachi, Pakistan; 2 Dr. Riaz Hussain Channa, MBBS. Department of Gastroenterology & Hepatology Jinnah Postgraduate Medical Center, Karachi, Pakistan; 3 Dr. Nazish Butt, MBBS, FCPS (Gastroenterology & Hepatology) Associate Professor and Head of Department, Department of Gastroenterology & Hepatology Jinnah Postgraduate Medical Center, Karachi, Pakistan

**Keywords:** Burden, Celiac disease, Malabsorption syndrome, Tuberculosis

## Abstract

**Objective:**

The objective of this study was to find out frequency and risk factors of malabsorption in patients presenting at tertiary care hospital, Karachi.

**Methods:**

This was a prospective-observational study conducted through a non-probability consecutive sampling technique. Ninety two adult patients presenting with a history of chronic diarrhea (diarrhea having duration of more than four weeks), age ≥14 years, both males & females, and diagnosed as malabsorption syndrome visiting out-patient or admitted in the department of Gastroenterology of the Jinnah Postgraduate Medical Center, Karachi between June 2018 and July 2020 were enrolled. Baseline and clinical data were recorded in a pre-designed questionnaire and analyzed using statistical package for the social sciences (SPSS) version 21.0.

**Results:**

The overall mean age and standard deviation of patient was 35.42±10.83 years. Diarrhea n=71 (77.17%), fever n=35 (38.04%), abdominal pain n=32 (34.78%), and weight loss n=13 (14.13%) were the most common symptoms observed in our study subjects. Most of the patients had normal upper GI endoscopy (26.56%) while multiple fundal erosions with pre-pyloric ulcer and severe pangastric erythema & classical scalloping of duodenal folds were most common findings observed, 21.87% and 17.18%, respectively.

**Conclusion:**

Our study provides evidence that malabsorption syndrome is most commonly present in males with younger age group and the most common causes were celiac disease and intestinal tuberculosis and most common presentation was diarrhea, fever, and abdominal pain.

## INTRODUCTION

Malabsorption syndrome is the result of disease(s) which affects small intestine usually, where most of the absorption takes place through microvilli and digestive enzymes or indigestion.^1^ Process of absorption affects when there is a disease in small intestine and its components such as microvilli.^2^-^4^ Malabsorption is usually a disease of developing nation where basic health facilities are hard to get. Millions of people in the world are currently living with malabsorption syndrome. Incidence and prevalence of malabsorption is greatly dependent upon the underlying etiology. The cumulative prevalence of malabsorption in United States is 1:133 and malabsorption caused by crohn’s disease is 20-100 per 100,000.^5^ In developed nations such as America, Germany, France, United Kingdom, and Australia gluten-sensitive enteropathy (GSE) is the most common cause of malabsorption and affects 1% of the patients while in Pakistan its actual burden in Pakistan is still not known.^6^,^7^

Determination of causative factors and associated clinical manifestations of malabsorption syndrome is crucial to understand and need to scientifically document. In Pakistan, the most common causes of malabsorption syndrome among adults 18 years and older are celiac disease, crohn’s disease, intestinal tuberculosis, and chronic pancreatitis.^8^ The actual burden and associated risk factors of malabsorption syndrome in Pakistan has not yet been studied some of the scattered data from small studies is insufficient,^9^,^10^ that is why this study aimed to determine the true burden of malabsorption syndrome in Pakistan and associated risk factors leading to this condition.

## METHODS

This was a prospective-observational study conducted through a non-probability consecutive sampling technique in which a total of 92 adult patients presenting with a history of chronic diarrhea (diarrhea having duration of more than four weeks), age ≥14 years, both males & females, and diagnosed as malabsorption syndrome visiting out-patient or admitted in the department of Gastroenterology of the Jinnah Postgraduate Medical Center, Karachi between June 2018 and July 2020 were enrolled. These included patients referred from multiple departments of the hospital and also from other hospitals of the Sindh province (as JPMC is the largest hospital of Sindh Province where patients come from all over the Pakistan).

### Exclusion Criteria:

Those patients who did not consent to participate, drug induced diarrhea, known case of lactose intolerance, and patients with diagnosed case of irritable bowel syndrome were excluded from this study.

### Ethical Approval:

It was taken from the hospital’s ethical committee before commencement of the study (Letter no. F.2-81/2019-GENL/10416/JPMC, dated: 01-02-2019).

Initially these patients were examined by the consultant who had an experience of at least five years in dealing such patients. A structured questionnaire was made to collect the relevant data such as patient’s baseline and clinical characteristics. Diagnosis of malabsorption syndrome was made based on the patient’s history, clinical manifestations, and clinical examination and then suspected disease was confirmed using gold standard available tests i.e. celiac disease was confirmed using anti-tissue transglutaminase antibody. We also performed upper and lower gastrointestinal endoscopy in patients’ whom these testes were indicated for the diagnostic purpose.

## RESULTS

A total of 92 patients were enrolled who met the inclusion & exclusion criteria. The overall mean age and standard deviation of patient was 35.42±10.83 years, most of them were married n=59 (64.13%), and belongs to urban areas n=62 (67.39%). Hypertension and Type-2 diabetes mellitus were the most common comorbids observed among our study participant, 29.34% and 25.0%, respectively. The frequency of betel nut chewers and gutka/pan were observed higher as compared to other addiction habits, 39.13% and 26.08%, respectively, Table-I. Mean levels of laboratory investigation were almost within normal limits except hemoglobin (9.07±6.12 gm/dL), white blood cell count (11.22±6.73 x 10^9^/L), estimated sedimentation rate (40.29±20.64/hour), and vitamin D3 levels (24.12±10.64/nmol/L), Table-II.

**Table-I:** Basic demographic and clinical characteristics of patients with malabsorption syndrome (N = 92).

Parameters		
Age - yeas (mean±SD)	35.42±10.83	
Male: Female ratio	68:60	

*Marital Status*	*N*	*%*

Single	29	31.52%
Married	59	64.13
Widowed	4	4.34
***Area of residence***		
Urban	62	67.39
Rural	30	32.6
***Comorbids***		
Diabetes Mellitus	23	25
Hepatitis B	2	2.17
Hepatitis C	2	2.17
Hypertension	27	29.34
Systemic Lupus Erythematosus	6	5.43
Rheumatoid Arthritis	3	3.26
Hypothyroidism	4	4.34
Hyperthyroidism	1	1.08
Human Immunodeficiency Virus	6	6.52
***Addiction***		
Alcohol	4	4.34
Smoking	18	19.56
Betel nut	36	39.13
Gutka/pan	24	26.08
Intravenous drug abuser	3	3.26

**Table-II:** Clinical investigations in patients with malabsorption syndrome (N = 92).

Parameters	Minimum	Maximum	Mean±SD
Hemoglobin - mg/dL	3	17.6	8.07±6.12
MCV – fl	62	106	82.1412.88
WBC - x109/L	2.3	21	11.22±6.73
Platelets x 109/L	22	450	127±100.62
ESR – hr	5	140	40.29±20.64
Urea - mg/dL	21	128	38.01±6.18
Creatinine - mg/dL	0.6	4.3	1.4±9.53
Sodium - mmol/L	109	141	127.01±7.53
Potassium - mEq/L	2.6	6.1	5.4±0.28
Chloride - mEq/L	94	107	100.21±5.42
Albumin - g/dL	2.4	5.1	3.49±0.88
Iron - mcg/dL	59	171	104.11±30.43
Calcium - mg/dL	7.8	10.2	7.01±1.82
Vitamin D3 - nmol/L	9.4	83	24.12±10.64

Symptomatology of patients diagnosed with malabsorption syndrome is shown in Fig-1. Diarrhea n=71 (77.17%), fever n=35 (38.04%), abdominal pain n=32 (34.78%), and weight loss n=13 (14.13%) were the most common symptoms observed in our study subjects.

**Fig-I:**
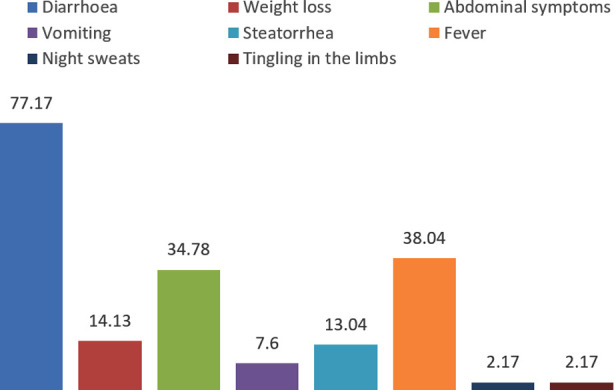
Clinical symptoms at the time of enrollment (N = 92).

Among all participants diagnosed with malabsorption syndrome, n=64 (69.56%) underwent esophagogastroduodenoscopy (EGD), n=58 (63.04%) underwent colonoscopy, and biopsies were performed among n=21 (22.82%) patients to determine the possible underlying cause of malabsorption syndrome and clinical symptoms. Most of the patients had normal EGD (26.56%) while multiple fundal erosions with pre-pyloric ulcer and severe pangastric erythema & classical scalloping of duodenal folds were most common findings observed, 21.87% and 17.18%, respectively. Ulcers in terminal ileum (20.68%) and ulcerated mucosa of cecum were most common colonoscopic findings (10.34%). The final diagnosis made through biopsy were tuberculosis and celiac diseases 57.14% and 23.8%, respectively. Table-II & Graph no. 2,3.

**Fig-II:**
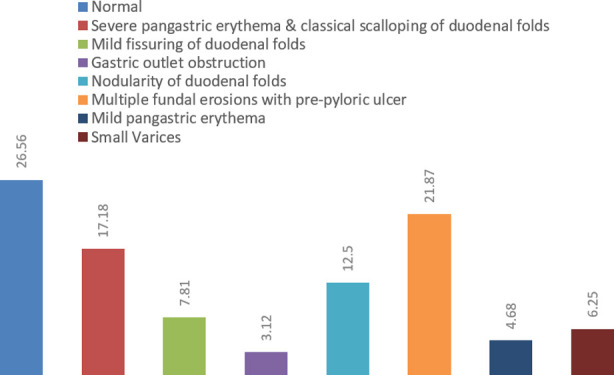
Upper gastrointestinal findings of patients with malabsorption syndrome (N = 64).

**Fig-III:**
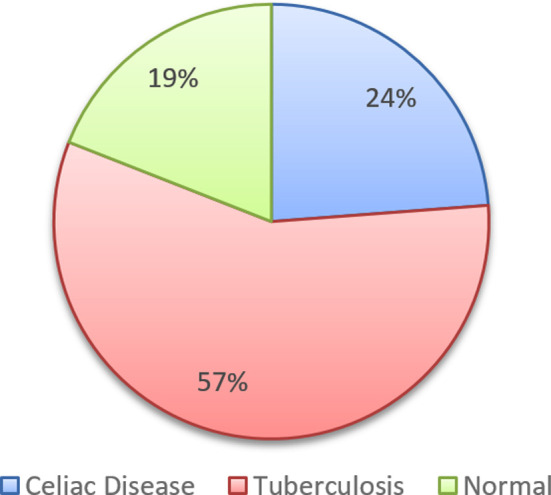
Post upper GI endoscopic, colonoscopy, and biopsy-based diagnosis in patients with malabsorption syndrome (N = 21).

## DISCUSSION

The most common cause of malabsorption syndrome in our study was presence of tuberculosis and celiac disease. In previously conducted most of the studies, celiac disease was the most common cause of malabsorption syndrome^4^,^11^-^13^ but most of these studies are from developed countries and not a single study is from developing country including Pakistan. One study from China has observed celiac disease and Whipple’s disease are the most common causes of malabsorption syndrome.^14^ While one study conducted in India has shown tuberculosis is one of the causes of malabsorption syndrome.^15^ Another study from India has shown tropical sprue, celiac disease, and crohn’s diseases were the most common causes of malabsorption syndrome.^16^

Malabsorption syndrome can affect all age groups depending upon the underlying cause. Like, in our study the most common age group was younger one and usually below the age of 40 years with male dominance. The most common symptoms patients presented were diarrhea, fever, abdominal symptoms, and weight loss. Previous studies suggest that symptoms in patients with malabsorption syndrome are solely depend upon the underlying cause such as patients with celiac disease, tropical sprue, and intestinal tuberculosis are most likely present with diarrhea and abdominal pain^1^,^17^,^18^, as shown in our study. The reason why diarrhea and abdominal pain were most common among our and previously published studies is scientifically the involvement of intestine which leads diarrhea and abdominal pain.

Low levels of hemoglobin are frequent presenting sign clinician observe at first instance. The most common cause of anemia are presence of iron or vitamin B12 deficiencies in our country. In a recently published study from Pakistan by the Khatoon S and colleagues has observed that significant proportion of patients (11%) with celiac disease had iron deficiency anemia (IDA) particularly in females.^19^ In another study, prevalence of IDA was reported between 1.8% and 14.6%. Therefore, duodenal biopsies should be taken during endoscopy if no obvious cause of iron deficiency (ID) can be found.^18^ In our study we also observed anemia in most of the patients. This could be because most of our study participants had intestinal tuberculosis and/or celiac disease.

Patients who presents with malabsorption syndrome should be evaluated for the cause. In patients, laboratory investigations insufficient to provide underlying cause of malabsorption syndrome, upper or lower gastrointestinal endoscopy should be performed along with tissue biopsies. Visual assessment of small intestine may reveal underling cause of malabsorption syndrome such as presence of villous shortening with crypt hyperplasia can be seen in patients with celiac disease and increased intraepithelial lymphocytes can be seen in patients with possible gluten-free enteropathy or viral gastroenteritis.^3^,^20^-^22^ Most of the patients had normal EGD (26.56%) while multiple fundal erosions with pre-pyloric ulcer and severe pangastric erythema & classical scalloping of duodenal folds were most common findings observed, 21.87% and 17.18%, respectively.

### Limitations:

Most importantly, the sample size is too small and the data were collected from a single center. The reason behind small sample size was less availability of patients diagnosed with malabsorption syndrome.

## CONCLUSION

Our study provides evidence that malabsorption syndrome is most commonly present in males with younger age group and the most common causes were celiac disease and intestinal tuberculosis and most common presentation was diarrhea, fever, and abdominal pain.
